# *Ginkgo biloba* Extract Attenuates Light-Induced Photoreceptor Degeneration by Modulating CAV-1—Redoxosome Signaling

**DOI:** 10.3390/antiox11071268

**Published:** 2022-06-27

**Authors:** Ke Wang, Yuan Chen, Xue Zhu, Wenjun Zou, Fanfan Zhou

**Affiliations:** 1 NHC Key Laboratory of Nuclear Medicine, Jiangsu Key Laboratory of Molecular Nuclear Medicine, Jiangsu Institute of Nuclear Medicine, Wuxi 214063, China; cyuan115@stu.njmu.edu.cn (Y.C.); zhuxue@jsinm.org (X.Z.); 2 Department of Radiopharmaceuticals, School of Pharmacy, Nanjing Medical University, Nanjing 211166, China; 3 Department of Ophthalmology, The Affiliated Wuxi No. 2 People’s Hospital of Nanjing Medical University, Wuxi 214002, China; 4 Sydney Pharmacy School, Faculty of Medicine and Health, The University of Sydney, Sydney, NSW 2006, Australia; fanfan.zhou@sydney.edu.au

**Keywords:** *Ginkgo biloba* extract, photoreceptor degeneration, white light illumination, CAV-1—redoxosome signaling

## Abstract

The clinical potential of Ginkgo biloba extract (GBE) in the prevention and/or treatment of retinal degenerative diseases has been widely explored; however, the underlying molecular mechanism is poorly understood. Photoreceptor degeneration is the hallmark of retinal degenerative diseases and leads to vision impairment or loss. In this study, the effect of GBE against white light (WL) illumination-induced photoreceptor degeneration was investigated, as well as its underlying mechanism. To evaluate the in vitro activity of GBE, analysis of cell viability, cell apoptosis, oxidative stress, NOX (NADH oxidase) activity and mitochondrial membrane potential (MMP), as well as Western blotting and transcriptome sequencing and analysis, were conducted. To evaluate the in vivo activity of GBE, HE staining, electroretinography (ERG), Terminal-deoxynucleoitidyl transferase (TdT)-mediated nick end labeling (TUNEL) assay and immunofluorescence analysis were conducted. Our study showed that GBE treatment significantly attenuated WL illumination-induced oxidative damage in photoreceptor 661W cells—a finding that was also verified in C57BL/6J mice. Further molecular study revealed that WL illumination downregulated caveolin-1 (CAV-1) expression, interrupted CAV-1-NOX2 interaction, re-located NOX2 from the cell membrane to the cytoplasm and induced the formation of redoxosomes, which led to cell death. However, these cytotoxic events were significantly alleviated by GBE treatment. Interestingly, CAV-1 overexpression showed a consistent protective effect with GBE, while CAV-1 silencing impacted the protective effect of GBE against WL illumination-induced oxidative damage in in vitro and in vivo models. Thus, GBE was identified to prevent photoreceptor cell death due to CAV-1-dependent redoxosome activation, oxidative stress and mitochondrial dysfunction resulting from WL illumination. Overall, our study reveals the protective effect of GBE on photoreceptors against WL illumination-induced oxidative damage in in vitro and in vivo models, which effect is mediated through the modulation of CAV-1-redoxosome signaling. Our findings contribute to better understanding the therapeutic effect of GBE in preventing photoreceptor degeneration in retinal degenerative diseases, and GBE may become a novel therapeutic agent that is effective in reducing the morbidity of these diseases.

## 1. Introduction

Photoreceptors, the first-order retinal neurons, are essentially involved in visual phototransduction, the dysregulation of which directly leads to impaired vision and/or blindness [1,2]. Photoreceptor degeneration is the main manifestation of many retinal degenerative diseases, such as retinitis pigmentosa (RP) [3] and late-stage age-related macular degeneration (AMD) [4]. Unfortunately, there are no proven therapies for photoreceptor degeneration; thus, prevention and/or slowdown of progression of photoreceptor degeneration are considered as the primary approach to reduce the morbidity of retinal degenerative diseases. It has been established that excessive light exposure is the main cause of photoreceptor degeneration [5]. Long-term blue light exposure may result in elevated oxidative stress and inflammation in the retinal pigment epithelium (RPE), increasing the risk of photoreceptor degeneration [6,7]. Currently, inducible retinal degeneration via light exposure has been widely adopted as the experimental approach in drug development against photoreceptor degeneration [8,9]. Since bright white light (WL) exposure induces a synchronized burst of photoreceptor degeneration in a large retinal area, it is considered better to mimic the natural environment that allows molecular exploration in a controlled fashion [10]. Oxidative stress is a primary consequence of WL-induced photoreceptor degeneration; to explore new agents that can protect photoreceptors from oxidative stress may be a clinical solution to treat retinal degenerative diseases [11,12].

It has been shown that oral administration of specific herbal medicines may delay the progression of retinal degenerative diseases [13,14]. *Gingko biloba* extract (GBE), derived from the leaves of *Gingko biloba*, is the most popular over-the-counter herbal medicine in the world due to its superior antioxidant activity [15,16]. GBE has been shown to be clinically active in the treatment of human neurological disorders, including Alzheimer’s disease [17,18], Parkinson’s disease [19], multiple sclerosis [20] and vertigo [21]. However, the clinical efficacy of GBE in the treatment of neurological diseases remains inconclusive. Many factors, such as population sensitivity, disease severity, assessments used to measure efficacy and doses, contribute to the variation in clinical outcomes [22,23]. In recent years, the neuroprotective effect of GBE in retinal degenerative diseases has been investigated in the laboratory as well as in clinical trials [24,25,26]. In a controlled double-blind trial, Fies and Dienel et al. have demonstrated that GBE treatment for over 6 months could markedly improve the vision of participants with dry senile macular degeneration [27]. It is well known that flavonoids, terpenes and several other components enriched in GBE possess antioxidative properties. Xie and Ranchon et al. have reported that intraperitoneal injection of GBE can protect the retina from oxidative injury and partially inhibit photoreceptor death [26,28]. However, the underlying mechanism of GBE’s cellular protective effect against light-induced photoreceptor degeneration is poorly understood. In this study, we extensively investigated the effect of extracts prepared from *Ginkgo biloba* dropping pills on photoreceptors against WL-induced oxidative damage in in vitro (661W cells) and in vivo (C57BL/6J mice) models.

## 2. Materials and Methods

### 2.1. Chemicals and Reagents

*Ginkgo Biloba* extract (GBE), prepared from *Ginkgo biloba* dropping pills, was kindly provided by Wanbangde Pharmaceutical Group Co., Ltd. (Wenling, China). Chemicals such as MTT (3-(4,5-dimethylthiazol-2-yl)-2,5-diphenyltetrazolium bromide), DAPI (dihydrochloride), mitoSOX, rhodamine123, NSC23766, VAS2870 and DMSO (dimethyl sulfoxide) were obtained from Sigma-Aldrich (St. Louis, MO, USA), ThermoFisher (Waltham, MA, USA) and MedChemExpress (Shanghai, China). The following antibodies were obtained from Santa Cruz Biotechnology (Dallas, CA, USA) or Abcam (Cambridge, MA, USA): Bax (cat. no. ab32503), Bcl-2 (cat. no. ab196495), Caspase-3 (cat. no. ab184787), CAV-1 (cat. no. ab32577), NOX2 (cat. no. sc-130543), p-SRC (cat. no. ab40660), SRC (cat. no. ab133283), p-Vav2 (cat. no. ab86695), Vav2 (cat. no. ab52640), Rac1 (cat. no. ab155938) and GAPDH (cat. no. sc-365062). The Rac1 activity assay kit (cat. no. STA-401-1) was obtained from Cell Biolabs (San Diego, CA, USA). Other chemicals and reagents used in the present study, unless otherwise specified, were obtained from Beyotime (Nantong, China) and Sangon (Shanghai, China).

### 2.2. Cell Line and WHITE Light Illumination

Cells from the 661W cell line were purchased from the American Type Culture Collection (ATCC, Manassas, VA, USA). The 661W cell line is a mouse cone photoreceptor cell line immortalized by expression of simian virus (SV) 40 T antigen (T-ag) driven by the human IRBP (interphotoreceptor retinoid-binding protein) promoter. Cells were cultured in Dulbecco’s Modified Eagle Medium (DMEM) supplemented with 10% (*v*/*v*) fetal bovine serum (FBS) and 1% penicillin–streptomycin (P/S) at 37 °C in a humidified atmosphere with 5% CO_2_. Cells were exposed to WL (450 nm) at a specific distance to maintain the light intensity at 4000 lux. WL was produced by a light-emitting diode white light source (OcuTech, Wuxi, China). For gene overexpression or silencing, pcDNA3.1-CAV-1 or CAV-1 siRNA was transfected using lipofectamine 2000 (Invitrogen, Waltham, MA, USA), according to the manufacturer’s instructions. Forty-eight hours after transfection, cells were harvested for molecular analysis.

### 2.3. Cell Viability and Apoptotic Assay

The MTT assay was used to determine cell viability. After treatment, MTT solution (0.5 mg/mL, 100 μL) was added to the cell culture, which was further incubated for 3 h at 37 °C. After removing the medium, DMSO (150 μL) was added to the cell culture for 10 min with gentle shaking. The absorbance was detected with a microstrip reader (Bio-Rad Laboratories, Hercules, CA, USA) at 490 nm wavelength. Annexin V-FITC and PI (propidium) double staining was used to determine cell apoptosis. After treatment, binding buffer (300 μL) containing 10 μL of Annexin V-FITC and 10 μL of PI was added to the cell culture and was further incubated for 15 min at 37 °C. The samples were then analyzed with flow cytometry (BD Biosciences, Franklin Lakes, NJ, USA).

### 2.4. Oxidative Stress and NOX Activity Analysis

DCFH-DA (2,7-dichlorodihydrofluorescein diacetate) staining was used to assess intracellular ROS generation. After treatment, DCFH-DA (10 μM) was added to the cell culture, which was further incubated for 15 min at 37 °C. The intracellular ROS fluorescence intensity was quantified by a fluorescence spectrophotometer (Molecular Device, San Jose, CA, USA). A H_2_O_2_ detection kit (titanium sulfate colorimetry assay) was used to assess intracellular hydrogen peroxide (H_2_O_2_) levels, according to the manufacturer’s instructions (Solarbio, Beijing, China). A NADH oxidase (NOX) colorimetric assay kit was used to assess NOX activity, with 2,6-dichlorophenol-indophenol (DCPIP) used as an artificial electron acceptor, according to the manufacturer’s instructions (Solarbio, Beijing, China).

### 2.5. Mitochondrial Membrane Potential Analysis

Rhodamine123 (Rh123) staining was used to assess mitochondrial membrane potential (MMP). This assay could measure mitochondrial membrane polarization in live cells. After treatment, cells were stained with rhodamine123 (0.5 mg/mL, 2 μL) for 30 min at 37 °C. The alternation of MMP level was determined with a fluorescence spectrophotometer (Molecular Device, San Jose, CA, USA).

### 2.6. Western Blot Analysis

Cells were lysed with RIPA (radioimmunoprecipitation assay) lysis buffer and a BCA (bicinchoninic acid) protein assay kit was used to assess protein concentrations. Protein samples (25 μg) were subjected to 12% SDS-PAGE gels and then transferred onto PVDF (polyvinylidene fluoride) membranes. The membranes were blocked with 5% non-fat milk in PBS-T (phosphate buffered saline–Tween20) and then incubated with each primary antibody (1:1000 dilution in PBS-T) at 4 °C overnight. Then, membranes were washed three times and incubated with secondary antibodies. The protein bands were detected using an ECL (efficient chemiluminescence) kit (Beyotime, Nantong, China). The density of each target protein was normalized to that of GAPDH.

### 2.7. Co-Immunoprecipitation Assay

The co-immunoprecipitation (Co-IP) assay was performed as previously described. Cells were homogenized in IP (immunoprecipitation) lysis/wash buffer. Then, the supernatants were collected upon centrifugation and added to anti-target antibody-cross-linked Protein A/G Plus agarose (100 μL in 1 mL protein supernatant) and then incubated at 4 °C overnight. Following that, nonspecific binding was eliminated by repeated washing with IP lysis/wash buffer. Protein samples pulled down with agarose beads were then eluted in 1× Laemmli buffer, with heating at 55 °C for 30 min. Eluted protein samples were then subjected to Western blot analysis using the indicated antibodies.

### 2.8. Transcriptome Sequencing and Analysis

RNA samples were extracted and prepared for transcriptome sequencing. The clustering of samples was processed through the cBot Cluster Generation System, according to the manufacturer’s instructions. After cluster generation, the library was sequenced using the Illumina Hiseq platform and 125 bp/150 bp paired-end reads were generated. HTSeq v0.6.0 was used to count the number of reads mapped to each gene. The FPKM (frangments per kilo base per million mapped reads) for each gene was calculated based on gene length, with read counts mapped individually. The transcriptome data have been uploaded to the NCBI BioSample database (accession numbers: SAMN28986237, SAMN28986238 and SAMN28986239). Genes with a fold-change of >2.0 and an adjusted *p*-value < 0.05 were assigned as differentially expressed genes (DEGs). The main functions of DEGs were analyzed by gene ontology (GO) analysis. The cluster Profiler R package (3.8.1) was used to test the statistical enrichment of DEGs in KEGG pathways.

### 2.9. Animal and White Light Illumination

Animal ethics approval was obtained from the Laboratory Animal Ethics Committee of Jiangsu Institute of Nuclear Medicine (Wuxi, China). Mice were raised in a 12 h light/dark cycle of 5 lux with free access to food and water. For experiments, age-matched mice (8–10 weeks old) were randomly assigned to three groups (n = 4 per group): non-light damage, white light damage (WL) and white light damage with GBE treatment (GBE). Mice in the WL group were exposed to 50,000 lux white light for 8 h per day/5 days (8 h/5 days), which protocol was optimized based on the previous report of Natoli et al. considering differences in spectral composition [29]. Mice of the GBE group were orally administered with GBE (100 mg/kg body weight/day) for 5 days and then exposed to 50,000 lux WL for 8 h/5 days with continuous GBE administration [30,31]. Pupils were dilated twice daily at 8 am and 1 pm with a single drop of 1% atropine sulfate (8.3 mg of atropine). For CAV-1 silencing, CAV-1 shRNA (AAV2-CAG-EGFP-mCAV-1-shRNA) was introduced in C57BL/6J mice. Briefly, mice were anesthetized and AAV2 construct-containing solution (final concentration of 1.8 × 1012 GC/mL) was administered (2 µL) through the sclera at a 45° angle into the vitreous. Animals were monitored and sacrificed at specified time points as indicated.

### 2.10. Electroretinography (ERG)

LabScribe v3.0 software using Ganzfeld (ERG 2, Phoenix Research Labs, Pleasanton, CA, USA) was used to record and analyze ERG response. After treatment, mice were subjected to ERG analysis. Anesthesia and pupil dilation were performed, and the eyes were kept moisturized using 0.5% hypromellose solution. Once the mice were sedated, the reference and ground electrodes were inserted subcutaneously into the head at the midline between the ears and tail, respectively. The positioning and alignment were performed according to the manufacturer’s instructions (Ganzfeld, ERG 2, Phoenix Research, Pleasanton, CA, USA). The procedure was conducted under normal light conditions (5 lux).

### 2.11. HE Staining and TUNEL Assay

The retinal tissues were removed from formalin and dehydrated using a series of increasing ethanol concentrations. Then, the tissues were cleared in xylene and embedded in paraffin blocks. For HE (hematoxylin and eosin) staining, paraffin sample sections (4 μm) were stained with hematoxylin (5 min) and then with eosin (2 min). All of the slides were mounted using neutral resin. For the TUNEL assay, sample sections were processed for TUNEL staining as previously reported [32]. In each region, the numbers of TUNEL^+^ cells were quantified in increments of 500 mm along the full length of the retina.

### 2.12. Immunofluorescence Analysis

Cells or tissues selected for immunofluorescence analysis were incubated in 10% normal goat serum for 1 h at 37 °C. Then, the samples were incubated with primary antibodies at 4 °C overnight. The samples were then washed and incubated with appropriate secondary antibodies conjugated with Alexa Fluor 488 or 594 for 4 h at room temperature. Visualization of immunofluorescence and image acquisition was performed using a Nikon A1 Confocal Microscope (Tokyo, Japan). DAPI (0.5 μg/mL) was used to stain the nuclei.

### 2.13. Statistical Analysis

Statistical analysis was performed using the SPSS 16.0 software package. All experiments were repeated for three independent replications. Data for multiple experiments are expressed as means ± SD. Statistical comparisons were conducted with the Student’s *t*-test between two groups and one-way ANOVA followed by Tukey’s post hoc test among three groups. *p* < 0.05 was accepted as statistically significant.

## 3. Results

### 3.1. GBE Attenuates Photoreceptor Degeneration in In Vitro and In Vivo Models Exposed to WL Illumination

**Figure 1:**
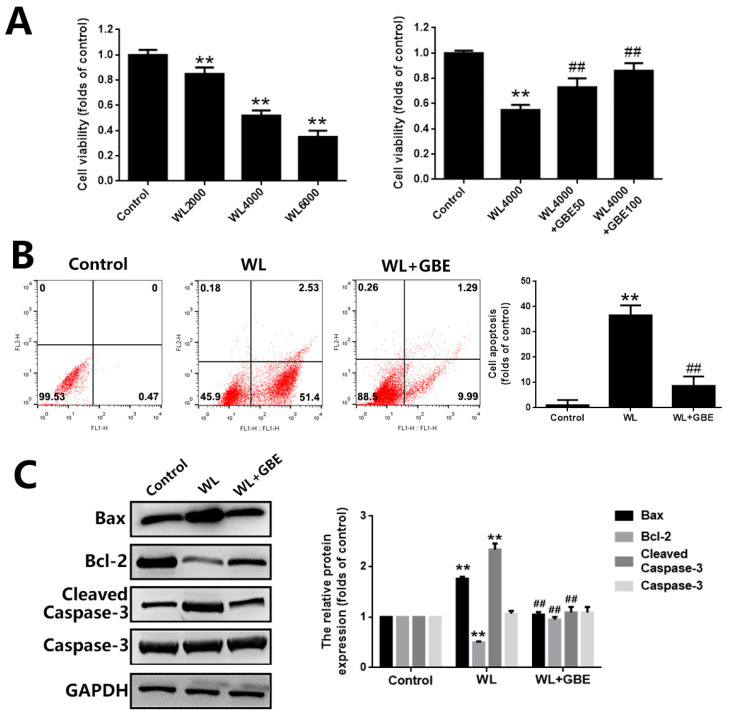
The effect of GBE on photoreceptor degeneration in 661W cells exposed to WL illumination. Cells from the 661W cell line pre-treated with GBE (50 or 100 mg/L, 24 h) were exposed to WL (4000 lux) for 2 h. (**A**) Cell viability upon WL treatment (2000, 4000 or 6000 lux) alone (left panel) and with GBE pre-treatment (right panel) was assessed by MTT assay. (**B**) Cell apoptosis was analyzed by Annexin V-FITC and PI double-staining assay. Representative images of cell apoptotic profiles are shown in the left panel and the percentages of apoptotic cells in each group are summarized in the right panel. (**C**) The expressions of apoptosis-related proteins (Bax, Bcl-2 and Caspase-3) were evaluated by Western blotting (left panel). The densitometry analysis of protein expression is shown in the right panel. ** *p* < 0.01 vs. Control, ## *p* < 0.01 vs. WL. WL: white light.

**Figure 2:**
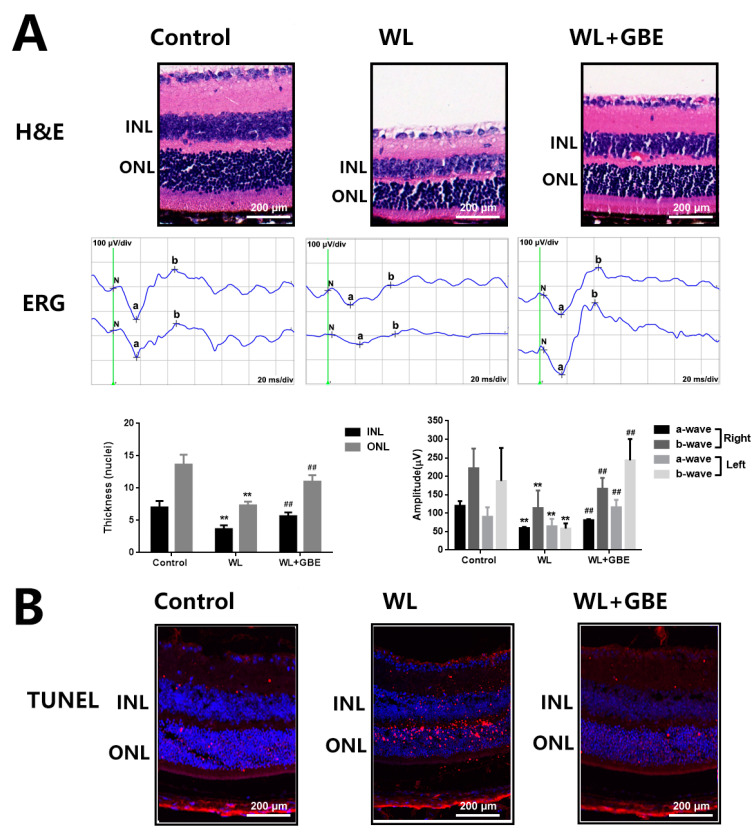
The effect of GBE on photoreceptor degeneration in C57BL/6J mice exposed to WL illumination. Mice were orally administered with GBE (100 mg/kg body weight/day for 5 days) and then exposed to WL (50,000 lux) for 8 h/5 days with continuous GBE treatment. (**A**) Retinal structure was illustrated by HE staining (upper panel) and retinal function was assessed by ERG analysis (middle panel). The thicknesses of the INL and ONL layers are summarized in the bottom-left panel and the amplitude of ERG is shown in the bottom-right panel. (**B**) Apoptosis of photoreceptors in retinal tissues was evaluated by TUNEL staining (TUNEL: red fluorescence, DAPI: blue fluorescence). ** *p* < 0.01 vs. Control, ## *p* < 0.01 vs. WL. WL: white light, ERG: electroretinography, INL: inner nuclear layer, ONL: outer nuclear layer.

To investigate the effect of GBE on photoreceptor degeneration induced by WL illumination, in vitro (661W cells—a mouse photoreceptor cell line displaying biochemical features of cone cells) and in vivo models (C57BL/6J mice) were adopted. Then, GBE pre-treatment was adopted for evaluating the preventive or early therapeutic effect of this drug in retinal degenerative diseases. In 661W cells, WL illumination (4000 lux, 2 h) significantly reduced cell viability (~0.5 folds of control) and induced cell apoptosis (~50 folds of control), which was accompanied by the increased expression of pro-apoptotic Bax protein and cleaved cell death mediator caspase-3 protein as well as reduced levels of anti-apoptotic Bcl-2 protein. The pre-treatment with GBE (100 mg/L, 24 h) attenuated WL-induced cytotoxicity and cell death and preserved the expression of the above-mentioned proteins (Figure 1). In mice, WL illumination (50,000 lux for 8 h/5 days) significantly impacted on retinal structure and function. HE staining showed that the inner and outer nuclear layers of the retinas in the WL-induced group became thinner and that cells in the retina were missing; however, GBE treatment with a concentration of 50 mg/kg/day and above could potently relieve these phenotypes in WL-exposed mice (data not shown). Thus, GBE at a concentration of 100 mg/kg/day was selected for the subsequent in vivo experiments. ERG analysis showed that a- and b-wave amplitudes of the WL-induced group were significantly decreased 0.5–2-fold, indicating the occurrence of photoreceptor dysfunction. The TUNEL assay demonstrated that photoreceptor apoptosis occurred in the WL-induced group. Importantly, GBE pre-treatment alleviated photoreceptor dysfunction and apoptosis in WL-exposed mice (Figure 2).

### 3.2. GBE Alleviated Redoxosome-Dependent Oxidative Stress and Mitochondrial Dysfunction

**Figure 3:**
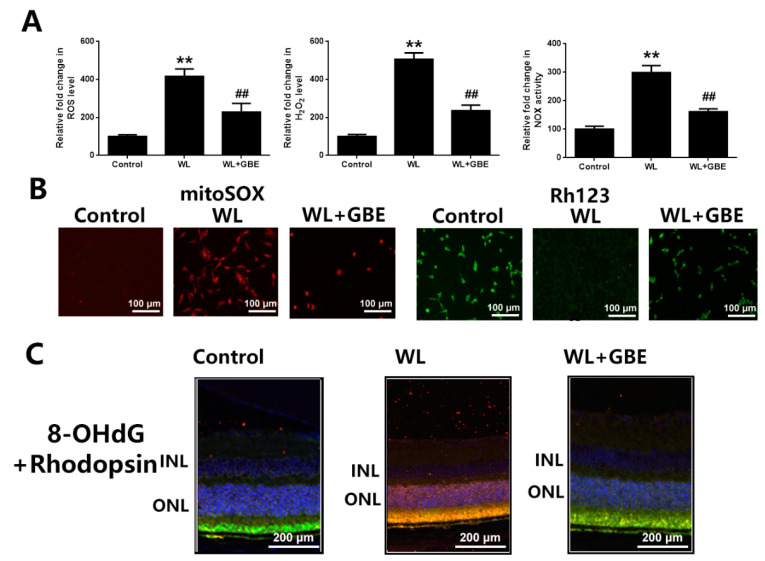
The antioxidative effect of GBE in 661W cells and C57BL/6J mice exposed to WL illumination. (**A**) Cells from the 661W cell line pre-treated with GBE (100 mg/L, 24 h) were exposed to WL (4000 lux) for 30 min. The level of intracellular ROS was assessed by DCFH-DA staining (left panel) and that of intracellular H_2_O_2_ was analyzed by titanium sulfate colorimetry (middle panel). The activity of NOX was evaluated using DCPIP assay (right panel). (**B**) The 661W cells pre-treated with GBE (100 mg/L, 24 h) were exposed to WL (4000 lux) for 2 h. The level of mitochondrial ROS was assessed using mitoSOX staining (left panel) and that of MMP was assessed using rhodamine123 staining (right panel). (**C**) Retinal tissues from mice with the indicated treatments were co-stained with DAPI (blue fluorescence), 8-OHdG (an ROS marker—red fluorescence) and rhodopsin (green fluorescence). ** *p* < 0.01 vs. Control, ## *p* < 0.01 vs. WL. WL: white light, DCFH-DA: 2,7-dichlorodihydrofluorescein diacetate, DCPIP: 2,6-dichlorophenol-indophenol, mitoSOX: mitochondrial superoxide indicator, MMP: mitochondrial membrane potential analysis, Rh123: rhodamine123, 8-OHdG: 8-Hydroxy-2′-deoxyguanosine, INL: inner nuclear layer, ONL: outer nuclear layer.

**Figure 4:**
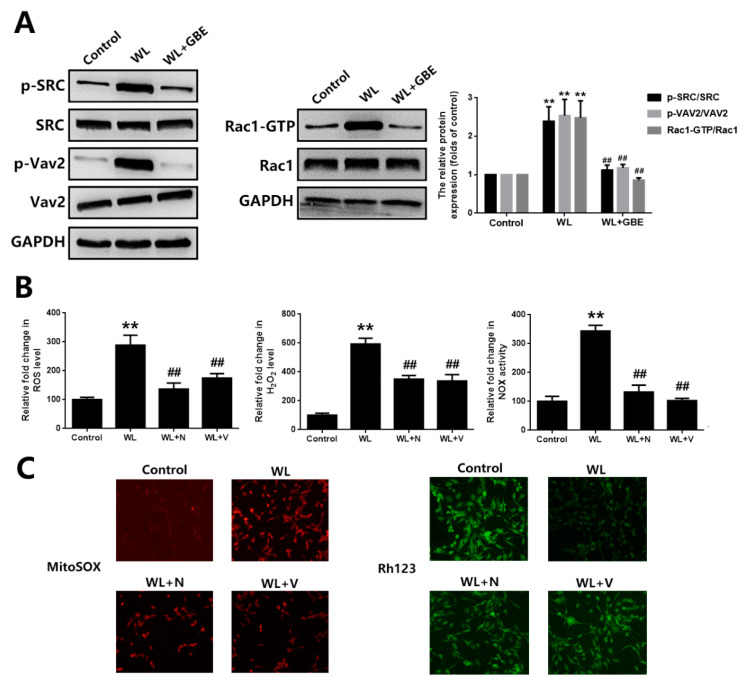
The influence of GBE treatment on redoxosome activation in 661W cells exposed to WL illumination. (**A**) Cells from the 661W cell line pre-treated with GBE (100 mg/L, 24 h) were exposed to WL (4000 lux) for 1 h. The expressions of redoxosome-related proteins were evaluated by Western blot analysis (left and middle panel). The densitometry analysis of protein expression is shown in the right panel. (**B**) The 661W cells pre-treated with Rac1 inhibitor (NSC23766, 80 µM, 6 h) or NOX inhibitor (VAS2870, 10 µM, 6 h) were exposed to WL (4000 lux) for 30 min. The level of intracellular ROS was assessed by DCFH-DA staining (left panel) and that of intracellular H_2_O_2_ was studied using titanium sulfate colorimetry (middle panel). The activity of NOX was evaluated using DCPIP as an artificial electron acceptor (right panel). (**C**) The level of mitochondrial ROS was measured with mitoSOX staining (left panel). The level of MMP was assessed by rhodamine123 staining (right panel). ** *p* < 0.01 vs. Control, ## *p* < 0.01 vs. WL. WL: white light, DCFH-DA: 2,7-dichlorodihydrofluorescein diacetate, DCPIP: 2,6-dichlorophenol-indophenol, mitoSOX: mitochondrial superoxide indicator, Rh123: rhodamine123, N: NSC23766, V: VAS2870.

To assess the molecular mechanism underpinning the cellular protective effect of GBE, oxidative stress and mitochondrial function analyses were conducted. Our data showed that WL illumination (4000 lux for 30 min or 24 h) significantly induced intracellular ROS, H_2_O_2_ generation and NOX activation and dysregulated mitochondrial function, as shown by increased mitochondrial ROS and decreased MMP in 661W cells. However, GBE pre-treatment (100 mg/L for 24 h) significantly attenuated WL-induced oxidative stress and mitochondrial dysfunction (Figure 3A,B). In mice, WL illumination (50,000 lux for 8 h/5 days) markedly upregulated the expression of 8-OHdG (ROS marker) in photoreceptors (rhodopsin staining); however, GBE treatment (100 mg/kg/day for 10 days) attenuated its expression in WL-exposed mice (Figure 3C). In addition, the role of redoxosomes in GBE’s antioxidant effect was evaluated. In 661W cells, WL illumination (4000 lux for 1 h) induced the phosphorylation of SRC tyrosine and Vav2 tyrosine, which, in turn, increased the expression of active Rac1-GTP and led to NOX activation. The pre-incubation of Rac1 inhibitor (NSC23766, 80 µM, 6 h) or NOX inhibitor (VAS2870, 10 µM, 6 h) could effectively protect the cells from WL-induced oxidative stress and mitochondrial dysfunction. Since GBE attenuated redoxosome activation, this suggested that GBE likely alleviated WL-induced oxidative stress and mitochondrial dysfunction in a redoxosome-dependent manner (Figure 4).

### 3.3. GBE Reduces Redoxosome Activation by Influencing the Interaction of CAV-1 and NOX2

**Figure 5:**
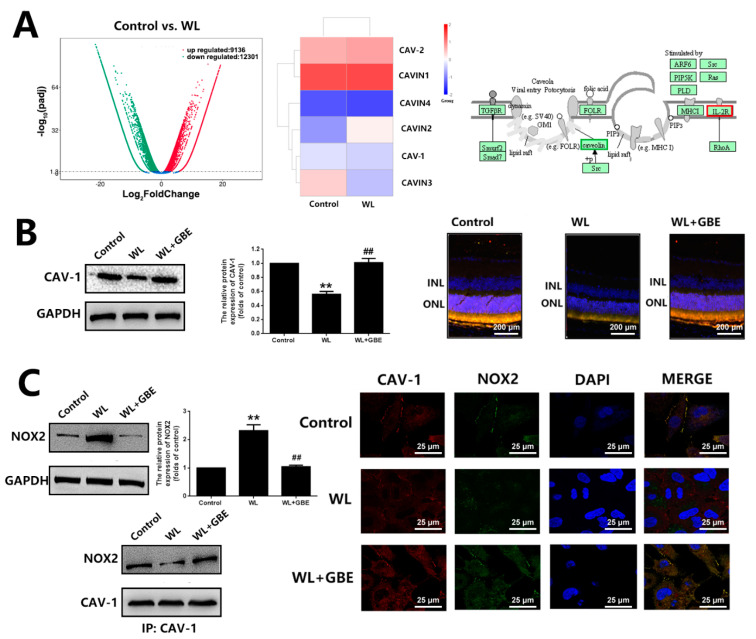
The influence of GBE on CAV-1 expression and CAV-1-NOX interaction in 661W cells and C57BL/6J mice exposed to WL illumination. (**A**) Transcriptome sequencing was performed on 661W cells pre-treated with or without GBE (100 mg/L, 24 h) exposed to WL (4000 lux) for 2 h. Volcano plots of the differentially expressed genes (DEGs) (left panel). Heatmap analysis of significantly changed caveolae-related genes (middle panel). KEGG pathway enrichment analysis of caveolae-related genes (right panel). (**B**) The 661W cells pre-treated with GBE (100 mg/L 24 h) were exposed to WL (4000 lux) for 2 h, and the expression of CAV-1 was assessed by Western blot analysis (left panel). The relative expression of CAV-1 is shown in the middle panel. Retinal tissues from mice with the indicated treatments were co-stained with DAPI (blue fluorescence), CAV-1 (red fluorescence) and rhodopsin (green fluorescence) (right panel). (**C**) The influence of GBE on NOX2 expression is shown in the left upper panel; the densitometry analysis of protein expression is shown in the right upper panel. CAV-1-NOX2 interaction was evaluated by co-immunoprecipitation (bottom-left panel). The protein lysate was pulled down by anti-CAV-1 antibody and then the samples were detected with anti-NOX2 antibody. The co-localization of CAV-1 (red fluorescence) and NOX2 (green fluorescence) was indicated with immunofluorescence staining (right panel). ** *p* < 0.01 vs. Control, ## *p* < 0.01 vs. WL. WL: white light, INL: inner nuclear layer, ONL: outer nuclear layer.

**Figure 6:**
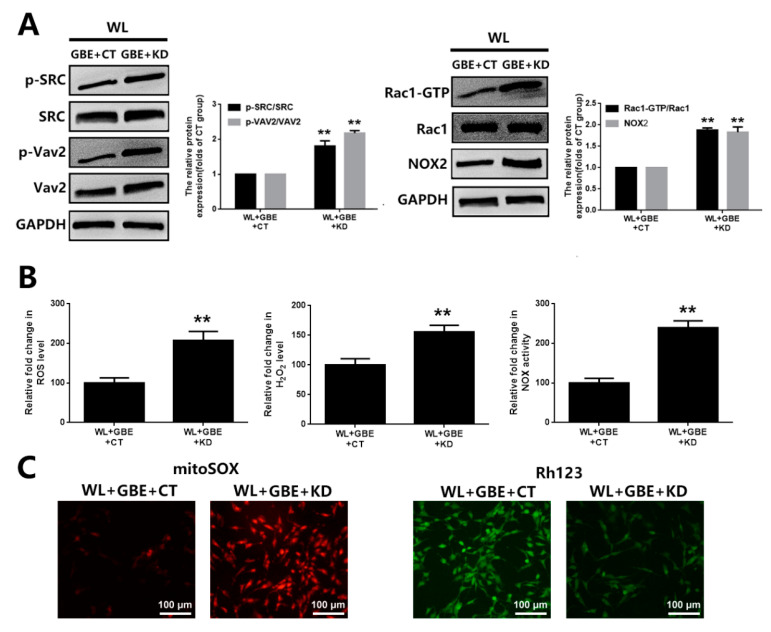
CAV-1 silencing attenuated the effect of GBE on redoxosome activation in 661W cells exposed to WL illumination. (**A**) Cells from the 661W cell line with or without CAV-1 silencing were pre-treated with GBE (100 mg/L, 24 h) and then exposed to WL (4000 lux) for 1 h. The expressions of redoxosome-related proteins were assessed by Western blot analysis. (**B**) The 661W cells with or without CAV-1 silencing were pre-treated with GBE (100 mg/L, 24 h) and then exposed to WL (4000 lux) for 30 min. The level of intracellular ROS was assessed by DCFH-DA staining (left panel). The level of intracellular H_2_O_2_ was assessed using titanium sulfate colorimetry (middle panel). The activity of NOX was assessed using DCPIP assay (right panel). (**C**) The level of mitochondrial ROS was assessed via mitoSOX staining (left panel) and that of MMP was assessed by rhodamine123 staining (right panel). ** *p* < 0.01 vs. WL + GBE + CT. WL: white light, DCPIP: 2,6-dichlorophenol-indophenol, mitoSOX: mitochondrial superoxide indicator, Rh123: rhodamine123 CT: 661W cells without CAV-1 silencing; KD: 661W cells with CAV-1 silencing.

To investigate the regulators involved in redoxosome activation, bioinformatic analysis and a co-immunoprecipitation assay were conducted. According to the bioinformatic analysis, there were 21,437 differentially expressed genes (DEGs) identified when comparing the WL group with the control group, among which, CAV-1 was significantly downregulated in both in vitro (4000 lux for 2 h) and in vivo models (50,000 lux for 8 h/5 days) exposed to WL. The co-immunoprecipitation assay showed that NOX2 was bound to CAV-1 in 661W cells, and the downregulation of CAV-1 in the WL-induced group was accompanied by the upregulation and relocation of NOX2 from the membrane to the cytoplasm as well as the formation and activation of redoxosomes (SRC-Vav2-Rac1-NOX). However, the pre-treatment with GBE (100 mg/L for 24 h) in WL-exposed cells (4000 lux for 2 h) reversed the altered expression of both proteins, as well as the relocation of NOX2, and subsequently blocked the formation of redoxosomes (Figure 5). In addition, CAV-1 silencing in 661W cells prohibited the suppressive effects of GBE on redoxosome activation, oxidative stress and mitochondrial dysfunction, suggesting that the impact of GBE on redoxosome activation was likely mediated by modulating the expression of CAV-1 and influencing the interaction of CAV-1 and NOX2 (Figure 6).

### 3.4. The Cytoprotective Effect of GBE on Photoreceptor Degeneration Is Exerted in a CAV-1 Dependent Manner

**Figure 7:**
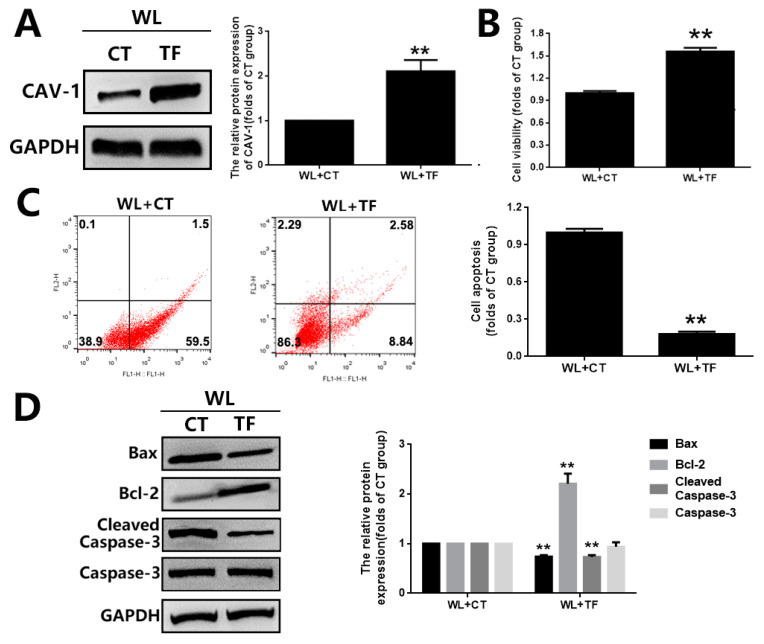
The effect of CAV-1 overexpression on photoreceptor degeneration in 661W cells exposed to WL illumination. (**A**) The overexpression of CAV-1 was verified by Western blot analysis. Cells from the 661W cell line overexpressing CAV-1 were exposed to WL (4000 lux) for 2 h. (**B**) Cell viability was assessed by MTT assay. (**C**) Cell apoptosis was analyzed by Annexin V-FITC and PI double-staining assay. Representative images of cell death profiles are shown on the left. The percentages of apoptotic cells are summarized in the right panel. (**D**) The expressions of apoptosis-related proteins (Bax, Bcl-2 and Caspase-3) were evaluated by Western blot analysis (left panel). The densitometry analysis of these proteins is shown in the right panel. ** *p* < 0.01 vs. WL + CT. WL: white light, CT: 661W cells without CAV-1 transfection, TF: 661W cells with CAV-1 transfection.

**Figure 8:**
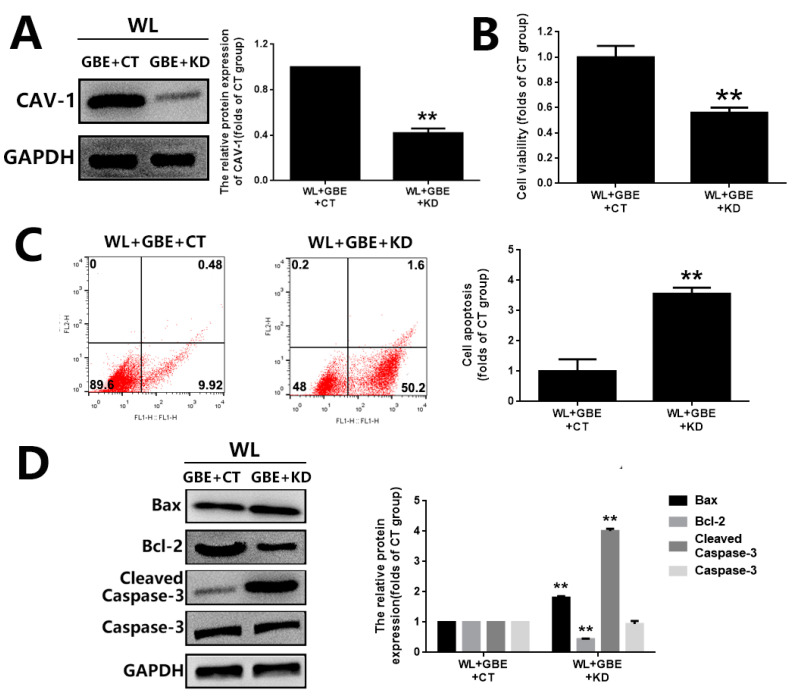
CAV-1 silencing attenuated the cytoprotective effect of GBE on photoreceptor degeneration in 661W cells exposed to WL illumination. The 661W cells with or without CAV-1 silencing were pre-treated with GBE (100 mg/L, 24 h) and then exposed to WL (4000 lux) for 2 h. (**A**) The silencing of CAV-1 was verified by Western blot analysis. (**B**) Cell viability was assessed by MTT assay. (**C**) Cell apoptosis was analyzed by Annexin V-FITC and PI double-staining assay. Representative images of cell apoptotic profiles are shown on the left. Percentages of apoptotic cells are summarized in the right panel. (**D**) The expressions of apoptosis-related proteins (Bax, Bcl-2 and Caspase-3) were evaluated by Western blot analysis (left panel). The densitometry analysis of each protein is shown in the right panel. ** *p* < 0.01 vs. WL + GBE + CT. WL: white light, CT: 661W cells without CAV-1 silencing, KD: 661W cells with CAV-1 silencing.

**Figure 9:**
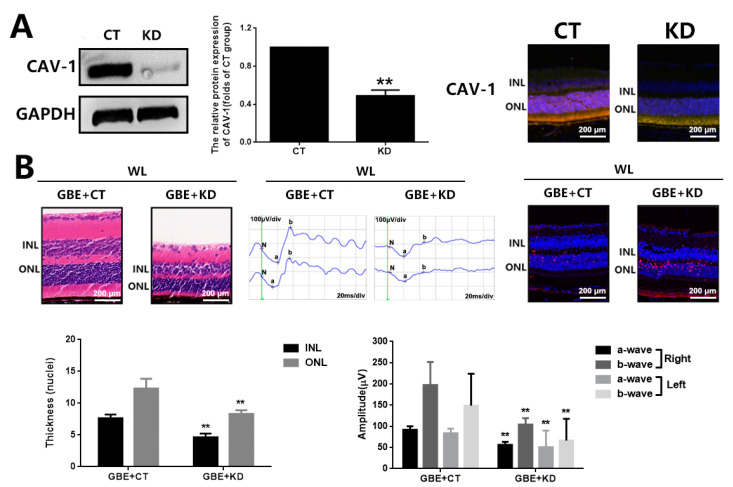
CAV-1 silencing attenuated the protective effect of GBE on photoreceptor degeneration in C57BL/6J mice exposed to WL illumination. (**A**) The silencing of CAV-1 in mouse retinas was verified by Western blot analysis (left and middle panels) and immunofluorescence staining (DAPI: blue fluorescence, CAV-1: red fluorescence, rhodopsin: green fluorescence) (right panel). (**B**) Mice with or without CAV-1 silencing were orally administered GBE (100 mg/kg body weight/day for 5 days) and then exposed to WL (50,000 lux) for 8 h/5 days with continuous GBE treatment. Retina structure was assessed by HE staining (left panel). Retina function was assessed by ERG analysis (middle panel). Photoreceptor apoptosis was assessed using TUNEL staining in the right panel (TUNEL: red fluorescence, DAPI: blue fluorescence). ** *p* < 0.01 vs. GBE + CT. WL: white light, INL: inner nuclear layer, ONL: outer nuclear layer, CT: Control, KD: CAV-1 silencing.

The involvement of CAV-1 in the antioxidative effect of GBE on photoreceptors was further evaluated in in vitro and in vivo models with CAV-1 overexpression or CAV-1 silencing. In 661W cells, CAV-1 overexpression and gene silencing was achieved with a CAV-1 expressing plasmid (pcDNA3.1-CAV-1) and CAV-1 siRNA transfection, respectively. CAV-1 overexpression significantly restored cell viability (~1.5 folds) and prevented cell apoptosis (~0.15 folds) in 661W cells exposed to WL, which was consistent with the effect of GBE (Figure 7). In addition, CAV-1 silencing significantly interfered with the cytoprotective effect of GBE in 661W cells exposed to WL (Figure 8). In mice, CAV-1 silencing was achieved by the transfection of AAV2-CAV-1 shRNA. The gene silencing of CAV-1 was verified by immunofluorescence staining and Western blot analysis. CAV-1 silencing in the mice prohibited the protective effect of GBE on photoreceptors, which induced pronounced retinal dysfunction and photoreceptor degeneration based on our assessment of retinal structure and function, as well as photoreceptor cell apoptosis, as indicated by HE staining, ERG analysis and TUNEL assay (Figure 9). These results indicated that GBE exerted its cytoprotective effect in a CAV-1-dependent manner.

## 4. Discussion

GBE has retinal protective effects due to its potential to increase blood flow and platelet-activating factor antagonism and prevent membrane damage caused by free radicals. The beneficial effect of GBE on vision has been reported in clinical trials with AMD patients [25]. Up to now, the mechanistic study of GBE in relation to retinal degenerative diseases has mainly relied on in vitro findings. For instance, a recent study investigated the antioxidant effect of 19 natural compounds isolated from GBE on human retinal epithelial pigmented (RPE) cells and found that rutin and procyanidin B2 are active compounds with potential therapeutic value in protecting RPE cells from oxidative injury [24]. However, there is a lack of in vivo evidence to verify the antioxidative effect of GBE and/or its active ingredients in retinal degenerative diseases.

Photoreceptor degeneration is a hallmark of retinal degenerative diseases and oxidative stress is one of its primary risk factors. Thus, in the present study, we assessed the protective effect of GBE on photoreceptors exposed to WL illumination in in vitro and in vivo models. As to the data reported in the literature and our preliminary testing, we found that 4000 lux in cells and 50,000 lux in mice were the optimal light densities for effectively inducing photoreceptor injury and these were adopted for model construction and GBE evaluation in the current study [29]. Noteworthy, the GBE used in our study is a previously verified preparation obtained through alcohol extraction from *Gingko biloba* dropping pills followed by purification with a macroporous resin column [33]. This preparation meets the standard quality indexes accepted worldwide: flavonoid glycosides ≥ 24%, terpene lactones ≥6%, ginkgolic acids ≤5 or 10 ppm [34]. We found that WL illumination significantly triggered photoreceptor degeneration and retina structure damage via induction of oxidative stress and mitochondrial dysfunction. More importantly, GBE pre-treatment could attenuate such phenotypes in 661W cells and mice exposed to WL (Figure 1, Figure 2 and Figure 3).

Caveolae, 50 to 100 nm flask-shaped invaginations of the plasma membrane, function in membrane trafficking, membrane lipid composition maintain and cell signal transduction [20]. CAV-1 is the major component of caveolae and is responsible for modulating a wide range of cellular events, such as proliferation, lipid metabolism, cellular tracking and signal transduction [35,36]. Recently, the role of CAV-1 in the retina has been widely investigated. Gu et al. reported that loss of CAV-1 causes blood–retinal barrier breakdown, venous enlargement and mural cell alteration [37]. Li et al. showed that loss of CAV-1 impairs retinal function due to disturbance of the subretinal microenvironment [38]. The important role of CAV-1 has also been identified in retinal degenerative diseases; however, its role in photoreceptor degeneration remains unclear. Our study showed that CAV-1 was significantly downregulated in in vitro and in vivo models exposed to WL, leading to photoreceptor degeneration. GBE pre-treatment could recover the impaired expression of CAV-1 (Figure 5). We identified that CAV-1 is highly expressed in the outer nuclear layer (ONL), outer segments (OS) and RPE in the retina, which is in contrast to the report of Li et al. [38]. Notably, Dean et al. revealed that CAV-1 is an authentic component of OS by subcellular fractionation [39], which may be because of the abundance of detergent-resistant membranes (DRMs) in OS, and CAV-1 is known to be one of the main components in DRMs. CAV-1 binds to numerous proteins via its scaffolding domain including NADPH oxidases (NOXs). In endothelial cells, CAV-1 is a negative regulator of NOX function by direct binding with NOX2 and NOX5 [40]. In human vascular smooth muscle cells, disrupting CAV-1 signaling triggers NOX-specific redox signaling and subsequent oxidative stress [41]. In addition, NOXs contribute to the formation and activation of redoxosomes, a fledgling area of cellular signaling through superoxide-producing endosomes. Redoxosome activation includes SRC kinase-dependent Vav2 tyrosine phosphorylation, Rac1-GTP activation and activation of NADPH oxidase [42]. In this study, CAV-1 downregulation in the WL group resulted in the upregulation and relocation of NOX2 from the membrane to the cytoplasm as well as the formation and activation of redoxosomes, which led to oxidative stress, mitochondrial dysfunction and photoreceptor cell apoptosis (Figure 4). However, GBE pre-treatment could significantly reduce the formation of redoxosomes via upregulating CAV-1 in in vitro and in vivo models (Figure 6), indicating that the antioxidative effect of GBE on photoreceptors against WL is involved in modulating CAV-1–redoxosome signaling. Thus, targeting CAV-1–redoxosome signaling might become a novel therapeutic target in the treatment of retinal degenerative diseases. Our further study revealed that the overexpression of CAV-1 could protect photoreceptors from oxidative damage resulting from WL exposure (Figure 7), while CAV-1 silencing largely prohibited the cytoprotective effect of GBE in both in vitro (Figure 8) and in vivo models (Figure 9). These findings confirmed the involvement of CAV-1 in the cytoprotective effect of GBE on photoreceptors against WL-induced oxidative damage.

Notably, the pharmacokinetic evaluation of GBE in mouse retinas has not been established at present and there is no accepted standard for the standardization of the plasma level of GBE yet. Future research is warranted to investigate ocular concentrations of GBE in systemic administration to indicate the therapeutic range of GBE in protecting photoreceptor degeneration; however, this is beyond the scope of the current study.

## 5. Conclusions

**Figure 10:**
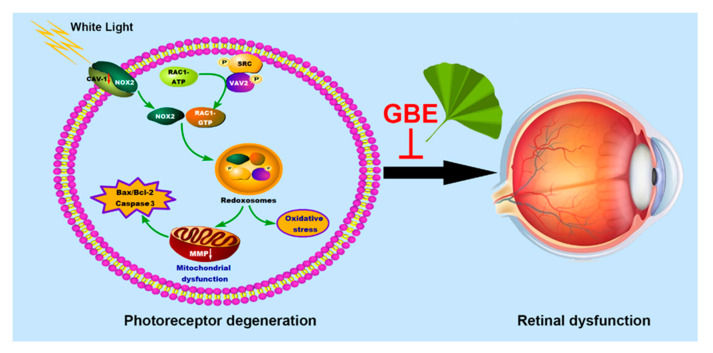
The proposed molecular mechanism of the effect of GBE on WL illumination-induced photoreceptor degeneration.

Overall, this study has demonstrated the neuroprotective effect of GBE against WL-induced photoreceptor degeneration in in vitro and in vivo models, which effect is dependent on CAV-1 and likely mediated through regulating its downstream redoxosome signaling (Figure 10). This pilot study provides critical information on the pathogenesis of photoreceptor degeneration leading to retinal degenerative diseases and forms a basis for potential clinical applications of GBE in preventing such diseases. In addition, future study is needed concerning the clinical effect of GBE on patients with retinal degenerative diseases.

## Data Availability

Data is contained within the article.
